# Quality gaps in care delivery among emerging adults with type 1 diabetes: A retrospective cohort study

**DOI:** 10.1002/hsr2.823

**Published:** 2022-10-03

**Authors:** Xinye S. Wang, Husayn Marani, Cheryl Harris‐Taylor, Leah Drazek, Janis Rusen, Nicola Farnell, Lorraine Lipscombe, Geetha Mukerji

**Affiliations:** ^1^ Faculty of Medicine University of Toronto Toronto Ontario Canada; ^2^ Institute of Health Policy, Management, and Evaluation, Dalla Lana School of Public Health University of Toronto Toronto Ontario Canada; ^3^ Women's College Hospital Institute for Health System Solutions and Virtual Care Women's College Hospital Toronto Ontario Canada; ^4^ Division of Endocrinology and Metabolism Women's College Hospital Toronto Ontario Canada; ^5^ Women's College Research Institute Women's College Hospital Toronto Ontario Canada

**Keywords:** emerging adult, nonattendance, transition of care, type 1 diabetes

## 1 INTRODUCTION

Emerging adulthood (EA), between 18 and 30 years of age, represents a high‐risk period for patients with type 1 diabetes mellitus (T1DM).^1^, ^2^, ^3^, ^4^ Loss‐to‐follow‐up rates (no appointment for >12 months) range from 25% to 40% up to years after discharge from pediatrics among T1DM EA patients,^3^, ^5^ which can compromise patient education and complication screening, and result in worsened glycemic control^6^, ^7^ and increased hospitalizations rates.^8^ This study aims to understand transition care quality gaps in an interdisciplinary T1DM EA clinic in a large Canadian city.

## 2 MATERIALS AND METHODS

This study was undertaken in an interdisciplinary T1DM EA (18–25 years) program at an academic, ambulatory hospital in Toronto, Canada, consisting of four endocrinologists, two nurses, one dietician, and one social worker.

A baseline audit of nonattendance rates of new and follow‐up T1DM EA was conducted between February 1, 2015 and September 30, 2016, from the hospital's electronic medical record system (Epic©). Nonattendance was defined as all missed scheduled appointments (no‐shows and cancellations <24 h) plotted monthly on a statistical P‐chart.

A retrospective chart review was conducted on all consecutive new patients referred to the EA program from February 1, 2015 to September 30, 2015. Information was collected for 1) demographic information, 2) attendance data, 3) glycemic/metabolic control, 4) routine screening (per Diabetes Canada Clinical Practice Guidelines [CPG]^9^), 5) acute diabetes complications, and 6) counseling documentation.^9^

Descriptive analyses were utilized with continuous variables reported as medians (interquartile range [IQR]) and categorical variables reported as percentages. *χ* ^2^ testing was used to compare categorical outcomes and Mann–Whitney *U* test to compare medians assessed for significance (*p* < 0.05).

## 3 RESULTS

### 3.1 Baseline nonattendance

**Figure 1:**
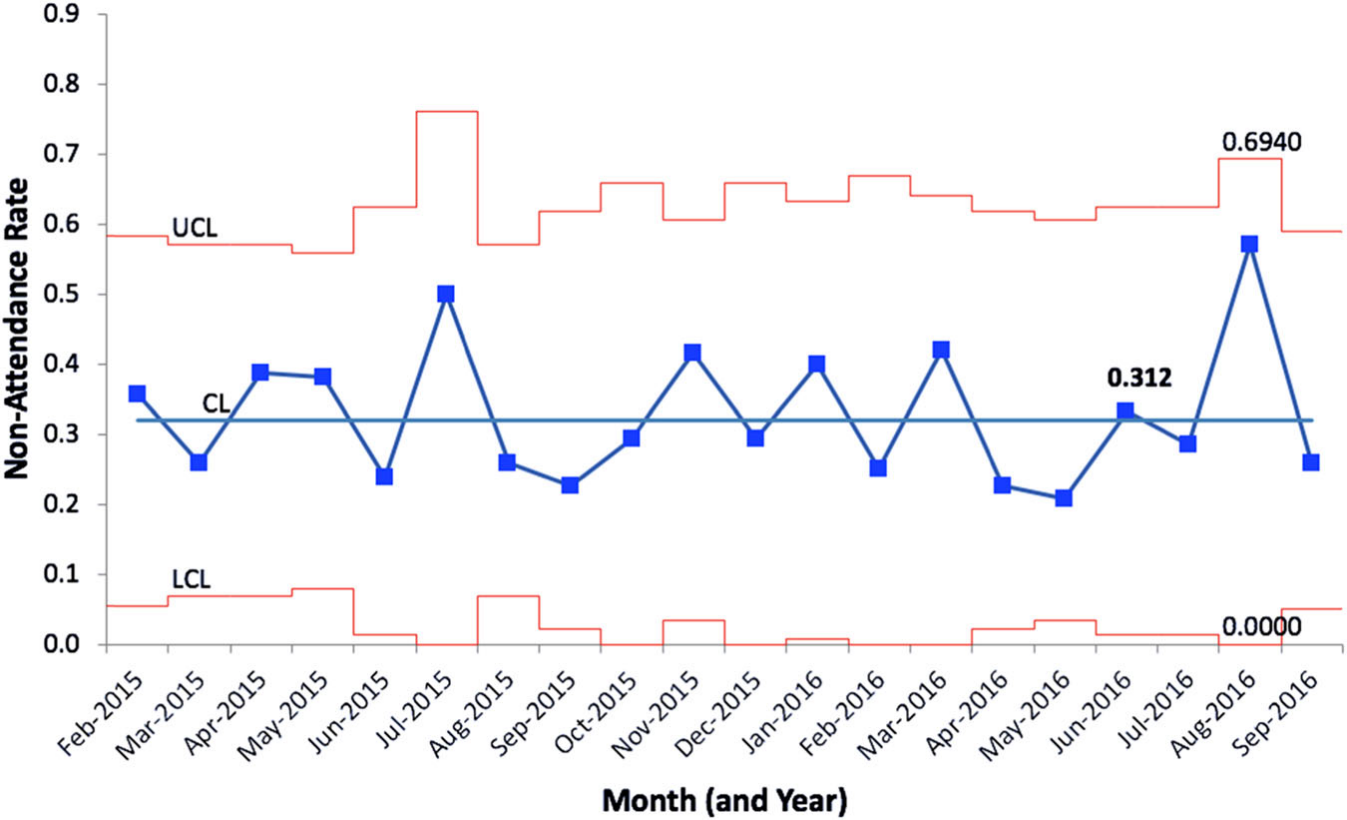
Statistical process control P‐chart depicting the baseline nonattendance rate of emerging adult patients at the Young Adult Diabetes Program. All prebooked in‐person clinical encounters were documented and the proportion of missed appointments to attended appointments was calculated to determine a nonattendance ratio per monthly basis. Missed appointments included both no‐show appointments and cancellations within less than 24 h. A total of 444 missed clinical encounters were registered for 150 patients over a 20‐month period. Upper control limit (UCL) = 0.694; center line (CL) = 0.312; and lower control limit (LCL) = 0.000. *y*‐axis = nonattendance rate (as a proportion of all in‐person clinical encounters); *x*‐axis = time (in months).

A total of 444 missed encounters were registered for 150 patients over 20 months. Mean nonattendance rate was 31.2% (Figure 1) with the no‐show rate (missed appointment without documented cancellation) at 23.7%.

### 3.2 Patient cohort demographics

Fifty‐one new referrals were registered between February 1, 2015 and September 30, 2015. The median age at the first visit was 22.9 years (IQR: 2.9 years) with 84.3% identifying female, and a median initial %HbA1c of 8.5% (IQR: 1.9%). Fourteen patients (27.5%) had comorbid autoimmune conditions and 11 (21.5%) had a pre‐existing psychiatric diagnosis. Nine patients (17.6%) had a previous severe hypoglycemic event and six (11.7%) had a previous diabetes‐associated hospitalization.

### 3.3 Attendance data

The baseline attendance rate of 51 new patients is presented in Table 1A. There were 13.7% of patients lost‐to‐follow‐up. Of those with follow‐up, the median number of visits per year per patient was 3.7, with a median of 2.7 visits with the endocrinologist. Overall, 35.3% of patients missed ≥2 appointments. Of those with ≥2 missed visits (nonattenders) compared to <2 missed visits (attenders), nonattenders had significantly lower median frequency of self‐blood‐glucose monitoring checks compared to attenders (2 vs. 3, respectively, *p* < 0.05), lower insulin pump use (45.5% vs. 5.6%, respectively, *p* < 0.05), and higher prior hospitalization rates for diabetes‐related complication (22.2% vs. 3%, respectively, *p* < 0.05) (Table 1B). There was no difference in age at the first visit, diabetes duration, %HbA1c, micro/macrovascular complications, and prior severe documented hypoglycemic events between nonattenders and attenders.

**Table 1A:** Clinical characteristics of new patients seen at the emerging adult type 1 diabetes clinic from February 1, 2015 and September 30, 2015 (*N* = 51)

*Patient demographics*	
Female	43/51 (84.3%)
BMI at the first visit	24 kg/m^2^ (IQR: 5)
Initial age at the first visit	22.9 years (IQR: 2.9)
Duration of diabetes at the first visit	8.2 years (IQR: 0.5)
Initial %HbA1C at the first visit	8.5% (IQR: 1.9)
On insulin pump at the first visit	16/51 (31.4%)
*Pre‐existing diabetes complications and medical comorbidities at the first visit*	
Presence of retinopathy	1/51 (2%)
Presence of microalbuminuria	0/51 (0%)
Presence of known neuropathy	1/51 (2%)
Known comorbid autoimmune conditions	14/51 (27.5%)
Confirmed prior psychiatric history	11/51 (21.5%)
Previous severe hypoglycemic event^a^	9/51 (17.6%)
Previous diabetes‐associated hospitalization^b^	6/51 (11.7%)
*Attendance data among new patients*	
% Lost to follow‐up^c^	7/51 (13.7%)
Duration in clinic (years)^d^ ^,^ ^e^	1.5 years (IQR: 0.4)
Visits per year^d^ ^,^ ^e^	3.7 visits/year (IQR: 2.6)
Endocrinologist or RN visits per year^d^ ^,^ ^e^	3.6 visits/year (IQR: 2.5)
Endocrinologist visits per year^d^ ^,^ ^e^	2.7 visits/year (IQR: 1.0)
% <2 nonattendance^f^	33/51 (64.7%)
% ≥2 nonattendance^f^	18/51 (35.3%)

Abbreviations: BMI, body mass index; EA, emerging adults; IQR, interquartile range; T1DM, type 1 diabetes mellitus; %HbA1c, glycated hemoglobin.

^a^ Defined as a hypoglycemic event requiring the assistance of another person to administer carbohydrate and/or glucagon, or to take other corrective actions.

^b^ Defined as any hospitalization resulting from a diabetes‐related complication (diabetic ketoacidosis and severe hypoglycemia) requiring third‐party assistance that occurred before the first visit at the EA transition clinic, and excludes hospitalization at the time of T1DM diagnosis.

^c^ Loss to follow up defined as a patient who has previously attended clinic but has not attended a follow‐up appointment in the clinic for over 12 months.

^d^ Values calculated based on the last value obtained in the data collection period.

^e^ Values are listed as median with the IQR listed in parentheses.

^f^ Nonattendance is defined as all no shows to appointments or cancellations within 24 h.

**Table 1B:** Clinical characteristics of patients stratified based on the frequency of nonattendance to visits between February 1, 2015 and September 30, 2015 (*N* = 51)

Nonattendance^a^	<2 Missed visits^a^ (*N* = 33) Attenders	≥2 Missed visit(s)^a^ (*N* = 18) Nonattenders	*p*‐Value
Age^b^	19 years (IQR: 3)	20 years (IQR: 2.5)	>0.05
BMI at first visit^b^	24 kg/m^2^ (IQR: 4.10)	22 kg/m^2^ (IQR: 4.33)	>0.05
Initial age at first visit^b^	19 years (IQR: 3)	19.5 years (IQR: 2)	>0.05
Duration of diabetes at first visit^b^	10 years (IQR: 6)	10 years (IQR: 4.5)	>0.05
Initial %HbA1C at first visit^b^	8.2% (IQR: 1.76)	9% (IQR: 2.76)	>0.05
Frequency of blood glucose monitoring at first visit^b^	3 checks/day (IQR: 1)	2 checks/day (IQR: 3)	**<0.05** *
Hypoglycemia frequency at first visit^b^	2 episodes/week (IQR: 1.6)	0.3 episodes/week (IQR: 0.7)	>0.05
On insulin pump at the first visit	15/33 (45.5%)	1/18 (5.6%)	**<0.05** *
Micro and/or macrovascular complications at first visit^c^	1/33 (3.0%)	3/18 (16.7%)	>0.05
Previous severe hypoglycemic event^d^	5/33 (15.2%)	3/18 (16.7%)	>0.05
Previous diabetes‐associated hospitalization^e^	1/33 (3.0%)	4/18 (22.2%)	**<0.05** *
Known comorbid autoimmune conditions (first visit)	8/33 (24.2%)	4/18 (22.2%)	>0.05
Confirmed prior psychiatric history (first visit)	6/33 (18.2%)	3/18 (16.7%)	>0.05

*Note*: *p*‐value (*p* < 0.05) derived from *χ* ^2^ analysis and Mann–Whitney *U* comparison.

Abbreviations: BMI, body mass index; EA, emerging adults; IQR, interquartile range; T1DM, type 1 diabetes mellitus; %HbA1c, glycated hemoglobin.

^a^ Nonattendance is defined as all no shows to appointments or cancellations within 24 h.

^b^ Values are listed as median with the IQR listed in parentheses.

^c^ Cumulative percentage of patients with pre‐existing microvascular (retinopathy, neuropathy, and nephropathy) or macrovascular (cardiovascular and cerebrovascular) complications at the first visit.

^d^ Defined as a hypoglycemic event requiring the assistance of another person to administer carbohydrates and glucagon or to take other corrective actions.

^e^ Defined as any hospitalization resulting from a diabetes‐related complication (diabetic ketoacidosis, severe hypoglycemia) requiring third‐party assistance that occurred before the first visit at the EA transition clinic, and excludes hospitalization at the time of T1DM diagnosis

* Denotes significance (bolded).

### 3.4 Preventative care and screening rates

Table 1C illustrates the frequency of CPG screening and counseling documented.^9^ Patients were consistent with regular %HbA1c checks at 6‐month intervals and annual eye screening (100% and 97%, respectively). Annual monofilament testing (69%) and lipid profile screening (67%) were inconsistent. Smoking status was documented 61% of the time. The frequency of documented counseling across 11 topics was variable. Preconception counseling, driving and hypoglycemia counseling, and hypoglycemia management counseling were most frequently discussed at 79.1% (*N* = 43, female patients), 70.6% (*N* = 51), and 68.6% (*N* = 51), respectively. The most infrequently documented counseling topics were medical alert counseling at 27.5% (*N* = 51), eating disorder screening at 21.6% (*N* = 51), and sick‐day management at 7.8% (*N* = 51).

**Table 1C:** Screening and counseling data from all new emerging adult patients referred to the young adult type 1 diabetes program between February 1, 2015 and September 30, 2015 (*N* = 51)

*Screening rates*	
% A1C, every 6 months	51/51 (100%)
% Eye exam, annual	49/51 (97%)
% TSH, annual	48/51 (94%)
% ACR, annual	44/51 (86%)
% Creatinine, annual	41/51 (81%)
% Monofilament test, annual	35/51 (69%)
% Lipid profile, annual	34/51 (67%)
% Smoking status documented	31/51 (61%)
*Documented counseling rates* a	
Preconception counseling	34/43 female patients (79.1%)
Driving and hypoglycemia counseling	36/51 (70.6%)
Hypoglycemia management counseling	34/51 (68.6%)
Mood screening	33/51 (64.7%)
Alcohol counseling	32/51 (62.7%)
Patient goal setting	30/51 (58.8%)
Smoking counseling	28/51 (54.9%)
Exercise counseling	24/31 (47.1%)
Medical alert counseling	14/51 (27.5%)
Eating disorder screening	8/51 (21.6%)
Sick day management counseling	4/51 (7.8%)

Abbreviations: ACR, albumin‐to‐creatinine ratio; TSH, thyroid‐stimulating hormone.

^a^ Counseling rates are documented as a pooled frequency of documented counseling topics covered across all clinical encounters over a 20‐month period.

## 4 DISCUSSION

We noted a nonattendance rate of 31%. Despite being structurally like other EA T1DM programs, our nonattendance rate was similar to general diabetes clinics and higher than the reported 12%–16% rate of other T1DM EA programs.^10^, ^11^, ^12^, ^13^ This may be due to cancellations <24 h being excluded from other nonattendance definitions. Higher nonattendance rates may result from a lack of a resource‐intensive transition coordinator.^11^, ^12^, ^14^, ^15^ Joint pediatric‐adult diabetes appointments have been effective for attendance,^12^, ^13^ but not feasible in Ontario where the pediatric cutoff age is provincially mandated. Our interdisciplinary team was only available during working hours, but some studies have benefitted from extended clinic hours for scheduling flexibility.^11^, ^14^ Although our EA program uses phone call reminders, email/text reminders were not permitted, which have been effective in improving attendance.^10^, ^12^, ^14^, ^15^ This suggests the need for context and resource‐specific interventions, and the need to address organizational barriers to streamline team communication.

Nonattenders had infrequent self‐blood‐glucose monitoring, were less likely to be insulin pump users, and had higher rates of diabetes‐associated hospitalizations compared to attendees. Nonattenders trended towards higher baseline %HbA1c values, although this was not significant. Similarly, in the literature, predictors of nonattendance include multiple‐daily injections, higher %HbA1c levels, and fewer physician visits before adult care.^15^ This suggests a need to identify high‐risk patients early and optimize diabetes management pretransition. Patients with closer monitoring and fewer complications were less likely to miss appointments in adult care.

Our study corroborates similar findings on variability in diabetes complication screening as a quality gap in transition care. An Australian study in a T1DM EA cohort^16^ showed only 12%–14% of patients having a documented ophthalmic examination, and 30.8%–32.6% had a documented albumin‐to‐creatinine ratio measurement over a 2‐year period.^16^ In our study, only 61% had a documented smoking screening assessment. One study reported only 30.4% of youth aged 10–14 years were asked about smoking, while only 47.2% were counseled on smoking cessation.^17^ Although screening guidelines have proven effective at informing clinical decision‐making, there continue to be application gaps. Nonattendance among T1DM EA may contribute to inconsistency in guideline application.

Overall, 21.6% of patients had documentation of eating disorder assessment in our study, indicating a need for more systematic screening of disordered eating; up to 10% of T1DM have an eating disorder.^18^ In our study, 79.1% of female patients received preconception counseling; this is important as a study demonstrated that knowledge of outcomes of uncontrolled diabetes and fetal development is unsatisfactory, but patient interest in receiving competent preconception education by a diabetologist is high (88.6%).^19^ Our study quantifies the rate of documented mood screening at 64.7%. A qualitative study of an Australian T1DM cohort (18–30 years) demonstrated little patient understanding of increased mental health risks.^20^ Our study reported documented sick day counseling, medical alert bracelet counseling, patient goal setting, and alcohol counseling rates at 7.8%, 27.5%, 58.8%, and 62.7%, respectively. To date, the frequency of these screening practices had not been reported.

Limitations in our study include a predominantly female and small patient cohort. Furthermore, our chart review represented a cohort that was receiving regular diabetes follow‐up; as such, there is underrepresentation from the population of patients who are loss‐to‐follow. We were unable to acquire data on diabetes‐related admissions or emergency room attendances between attenders and non‐attenders.

## 5 CONCLUSION

This study highlights important quality gaps in transition care delivery for T1DM EA. Future studies involving the implementation of patient‐centered interventions, while being mindful of local contextual factors and resources, will be an important way of improving care delivery of T1DM EA.

## AUTHOR CONTRIBUTIONS

**Geetha Mukerji**: Conceptualization, resources, and supervision. **Xinye S. Wang**: Data curation and investigation. **Xinye S. Wang and Husayn Marani**: Formal analysis and visualization. **Geetha Mukerji and Xinye S. Wang**: Methodology. **Xinye S. Wang, Geetha Mukerji, and Husayn Marani**: Writing–original draft preparation. **Cheryl Harris‐Taylor, Leah Drazek, Janis Rusen, Nicola Farnell, and Lorraine Lipscombe**: Writing–review and editing. All authors have read and approved the final version of the manuscript.

## CONFLICT OF INTEREST

The authors declare no conflict of interest.

## ETHICS STATEMENT

The study protocol was reviewed and approved by the Women's College Hospital Ethics Assessment Process for Quality Improvement Projects.

## TRANSPARENCY STATEMENT

The lead author Geetha Mukerji affirms that this manuscript is an honest, accurate, and transparent account of the study being reported; that no important aspects of the study have been omitted; and that any discrepancies from the study as planned (and, if relevant, registered) have been explained.

## Data Availability

Data are available upon request by contacting the corresponding author, Dr. Geetha Mukerji. Dr. Geetha Mukerji had full access to all of the data in this study and takes complete responsibility for the integrity of the data and the accuracy of the data analysis.
